# Making ward-based outreach teams an effective component of human immunodeficiency virus programmes in South Africa

**DOI:** 10.4102/sajhivmed.v19i1.778

**Published:** 2018-04-12

**Authors:** Nireshni Naidoo, Jean Railton, Geoffrey Jobson, Nthabiseng Matlakala, Gert Marincowitz, James A. McIntyre, Helen E. Struthers, Remco P.H. Peters

**Affiliations:** 1 Anova Health Institute, Johannesburg & Tzaneen, South Africa; 2 School of Public Health, University of the Witwatersrand, South Africa; 3 Mopani DCST, Department of Health, Limpopo Province, South Africa; 4 School of Public Health & Family Medicine, University of Cape Town, South Africa; 5 Division of Infectious Diseases & HIV Medicine, Department of Medicine, University of Cape Town, South Africa

## Abstract

The implementation of ward-based outreach teams (WBOTs), comprised of community health workers (CHWs), is one of the three interventions of the South African National Department of Health’s (NDoH) Primary Health Care (PHC) Re-engineering strategy for improving health outcomes. CHWs provide a necessary structure to contribute to successful implementation of the human immunodeficiency virus (HIV) programme in four ways: (1) prevention of HIV infection by health education, (2) linkage to care by health education and referrals, (3) adherence support and (4) identification of individuals who are failing treatment. However, CHW programme and HIV programme-specific barriers exist that need to be resolved in order to achieve maximum impact. These include a lack of stakeholder and community support for WBOTs, challenging work and operational environments, a lack of in-depth knowledge and skills, and socio-cultural barriers such as HIV-related stigma. Considering its promising structure, documentation of the WBOT contribution to healthcare overall, and the HIV programme in particular, is urgently warranted to successfully and sustainably incorporate it into the South African healthcare system.

## Introduction

Ward-based outreach teams (WBOTs) are becoming an increasingly important part of primary healthcare (PHC) in resource-constrained rural settings across the globe.^1^ In addition to district clinical specialist teams and integrated school health services, WBOTs are one of the three components of the PHC re-engineering programme introduced in South Africa in 2012 to improve communities’ access to health services and the quality of care provided.^2^ In South Africa, each WBOT is linked to a PHC facility and consists of a team leader, usually a professional nurse, plus five or more community health workers (CHWs). Each CHW is allocated 250–400 households to support.^3^

Several approaches have been initiated to improve outcomes of the HIV programme in South Africa, including human immunodeficiency virus (HIV) counselling and testing and adherence clubs.^4^ Specifically, these approaches have been either community-based or facility-based. WBOTs provide a means of bridging the gap between health facilities and the communities they serve, a role which may be especially valuable in rural areas where communities are impoverished and under-served, and where individuals have to travel excessive distances to seek medical care.^1^ CHW’s primary activities include registration of household’s health needs, provision of health education and the provision of adherence support for individuals on antiretroviral therapy (ART) or with other chronic illnesses. In this article, we identify and discuss key opportunities for effectively integrating WBOTs in the HIV response in South Africa, while noting various challenges that need to be addressed in order to maximise impact.

## Key opportunities for ward-based outreach teams in South Africa’s human immunodeficiency virus programme

The WBOTs are well positioned at the interface of the community and facility to contribute to the HIV programme in the following ways (Table 1):

- provision of information to educate individuals on how to prevent transmission of HIV infection
- identification of individuals at risk for HIV infection who should be tested for HIV
- provision of adherence support and tracing of individuals with missed appointments to improve retention in care
- early identification of individuals with deteriorating health while on ART to reduce further morbidity and mortality.

**TABLE 1:** Summary of potential contributions of WBOTs to the HIV programme

Opportunities	Activities of WBOTs	Expected outcomes
1. Provision of information to educate individuals on how to prevent transmission of HIV infection	Educate community about HIV prevention during household visits	Reduction in newly HIV-infected individuals
2. Identification of individuals at risk for HIV infection who should be tested for HIV	Re-educate individuals about HIV Refer individuals to the PHC facility for HIV testing	Increased uptake of HIV testing and ART initiation
3. Provision of adherence support and tracing of individuals with missed appointments to improve retention in care	Refer defaulting individuals back to the PHC facilities Implement adherence health clubs to support ART maintenance for groups of HIV-infected stable individuals	Reduction in number of individuals defaulting in treatment taking
4. Early identification of individuals with deteriorating health while on ART to reduce further morbidity and mortality	Refer individuals with deteriorating health due to chronic diseases to the clinic for treatment Distribute medication to bedridden patients	Reduction in mortality and morbidity of HIV-infected individuals with deteriorating health and chronic diseases

WBOT, ward-based outreach teams.

### Prevention of human immunodeficiency virus infection and its complications

There is evidence showing the effectiveness of using CHWs in large-scale health promotion and disease prevention programmes in low-income countries such as Haiti, Ethiopia, Uganda and Malawi.^5,6^ This preventative aspect of CHWs’ work could also play a critical role in South Africa’s HIV programme. CHWs could support the programme specifically not only by providing health education on how to prevent HIV infection but also by distributing condoms and potentially assisting in the identification of individuals that may benefit from pre-exposure prophylaxis (PrEP).^7,8,9,10^ CHWs, through adherence support, could contribute to lowering community viral load (VL) and thereby reducing transmission in the community.^11,12^ However, it has not yet been documented how well the above roles are performed and whether CHWs’ efforts positively impact on the reduction of HIV incidence. To our knowledge, there are so far no South African studies undertaken that assess the impact of preventative aspect of CHWs’ work.

### Identification of human immunodeficiency virus-infected individuals

CHW’s link between communities and facilities makes them well placed to support and appropriately refer vulnerable individuals to health facilities. Amongst those classified as vulnerable by the National Department of Health (NDoH) are individuals at risk for HIV infection and those already infected.^3^ As such, discussion of HIV status and HIV risk with individuals, their partners and children is an important component of CHW activity. South Africa is one of the first countries to formally adopt the Universal Test and Treat approach recommended in the updated World Health Organization (WHO) guidelines on HIV treatment. The guidelines state that all HIV-infected individuals should start ART within two weeks of an initial CD4 count being performed.^13^ There are limited data about the feasibility of HIV case identification by CHWs in South Africa, but a study by Uys, conducted at seven sites in South Africa, shows that community caregivers who provide psychosocial and household support may have a positive impact on HIV case identification through mobilisation and referral of individuals to HIV services for testing.^14^ CHWs, on the contrary, focus on providing medical aspects of care. Although this study was conducted in the setting of a home-based AIDS care model, and community caregivers are not the same as CHWs, the results are encouraging and support the involvement of a structure such as WBOT that bridges the gap between the community and the healthcare facility by complementing services provided by community caregivers.^15^

CHWs could also make an important contribution to the HIV response by supporting home-based HIV testing. This approach to HIV testing and counselling was used in a cluster randomised controlled trial in KwaZulu-Natal Province, South Africa, which found significantly higher rates of HIV testing amongst participants in the home-based testing arm than those testing at facilities (69% vs. 47%; 95% CI 1.32–1.81). Other results included a higher uptake of couple counselling and testing as well as a reduction in multiple partners.^16^ In light of this, to strengthen the impact of CHWs in the HIV programme, home-based testing by CHWs should be considered. There is also potential for HIV testing during community-based campaigns^17^ and HIV self-testing as recommended by the WHO.^18^

### Tracing non-adherent individuals and provision of adherence support to improve retention in care

A high level of patient retention on antiretroviral treatment is imperative for good HIV programme outcomes. CHWs can improve retention in care directly by tracing individuals who missed their appointments or defaulted treatment and linking them back into care at the healthcare facility. Although tracing is an important part of the CHW’s activities, the effectiveness of this approach remains undocumented. Defaulter tracing should not become so time consuming that it prevents CHWs from effectively undertaking their other duties. In the South African WBOT programme, we suggest that defaulter tracing should combine an initial attempt to contact non-adherent individuals telephonically, followed by physical tracing at their homes by CHWs if they are not reachable via telephone. This was shown to be successful in other studies^19^, and preliminary data from Mopani District, Limpopo Province, are promising, suggesting that there is value in using a combination of telephone and physical tracing as a routine CHW activity (R.P. Peters, 2017, n.d., personal communication).

Another way in which CHWs could contribute to improving retention in care is through adherence support. Grimwood et al.,^20^ in a multicentre cohort study, demonstrated that community-based adherence support is an effective way to improve patient retention on ART. In that study, patient advocates (PAs) were tasked with providing adherence support to the community. Clinical, virological and immunological outcomes were compared between children who did and did not receive community-based adherence support from PAs in Western Cape, KwaZulu-Natal, Eastern Cape and Mpumalanga provinces. They showed a significant difference in patient retention after three years: 92% (95% CI: 76.7% – 97.6%) amongst children with PAs and 74% (95% CI: 65.4% – 81.0%) amongst children without PA support. In addition, multivariable analyses showed that children with PAs had reduced probability of HIV-related programme attrition and mortality.^20^ Similarly, Igumbor et al.^12^ found that a significantly higher proportion of patients (70% vs. 30%) with a community-based adherence supporter had a VL of less than 400 copies/mL at six months of treatment.^12^ In the context of the WBOT programme, adherence support entails regularly visiting patient’s households to supervise medication taking, educating patients about the importance of taking treatment exactly as prescribed by the healthcare workers, provision of tips and tools such as diary record cards to aid in timely treatment taking and referrals of non-adherent individuals to the nearest PHC facility for follow-up.

The positive value of adherence support in retaining individuals on ART in South Africa was also documented in the study by Igumbor et al.,^12^ which evaluated the effect of community adherence support on ART programme retention. They observed that a significantly higher proportion of patients with a community-based adherence supporter (89%) had a treatment pick-up rate of over 95%.^12^ Adherence support by CHWs may also play an important role in reducing health facility workloads through monitoring and supporting HIV-infected patients who are stable on ART.^21,22,23^ Grimsrud et al.,^4^ for example, found that task-shifting to CHWs can successfully support and improve ART adherence and encourage patient self-management.^4^ Over a period of 12 months, more than 2000 stable ART patients were successfully decentralised from a doctor-driven PHC clinic to a community-based model of care including CHWs, while maintaining ART retention rates of 97% after six months and 94% at 12 months.^4^ The ongoing scale-up of the ART programme in South Africa, particularly as test and treat becomes routine, makes it increasingly important to find effective ways to involve CHWs in novel systems of alternative distribution of ART and long-term ART retention, especially in resource-limited settings.

### Early identification of individuals failing treatment

Finally, CHWs have a critical role to play in maintaining HIV viral suppression and hence reducing HIV-related morbidity and mortality through ongoing monitoring of individuals on ART. In this role, CHWs essentially provide a means for the early detection of individuals at risk of defaulting or treatment failure through symptom screening. By enhancing treatment literacy, early identification of ART side effects, awareness of psychosocial and poverty-related factors that may impact the likelihood of patient’s virological failure, CHWs can be an essential link in the HIV treatment cascade. Grimwood et al.^20^ demonstrate this potential by finding that the presence of community-based patient support reduced mortality of children after three years of ART: 3.7% of children died in a setting with PAs, whereas 8.0% of children died without community-based support.^20^

## Barriers and challenges to successful ward-based outreach team support

Despite the promise of the WBOTs in improving programme outcomes, there are several key barriers and challenges to their successful implementation in South Africa’s healthcare system. These include general operational challenges and HIV programme-specific challenges as discussed below.

### General challenges

#### Varying perceptions of community health worker roles

The health system identifies CHWs as volunteers who manage patients within the community in which they reside and usually receive a stipend for their services.^24^ Some community members perceive CHWs to be peers, while others are knowledgeable about CHW roles in communities such as promoting primary health services and encouraging testing for several health conditions.^25^ CHWs generally perceive themselves as being a part of the community, while providing services and making a difference to the lives of people. Some even have an elevated perception of their roles as being teachers, social workers and doctors as they are given access to privileged medical information of patients and are linked to the formal health system.^26^ However, this empowerment may be challenged by difficult working environments. A recent multi-country comparative study shows that CHWs are often left feeling unsupported and undervalued as organisational and relational challenges hindered their work. Therefore, improving organisational support and helping the community better understand and appreciate the roles of CHWs could possibly mitigate these challenges faced by CHWs.^27^

#### Lack of stakeholder and community support

A fundamental challenge that limits the effectiveness of WBOTs in South Africa is their poor integration into local health systems, which in turn undermines the role of CHWs in community healthcare.^7^ Effective integration of WBOTs requires PHC facilities in the CHW programme to adopt a coordinating role^28^ and to facilitate ongoing contact with CHWs for the purposes of feedback, training and monitoring. A study in Uganda, for example, found that creating a bidirectional feedback loop by holding monthly meetings and using data collected timeously improved CHW activities and ensured that the continuum of services from community to facility is maintained.^29^ The integration of CHWs into a well-established community-based PHC system with facility nurses, doctors, a proper referral system and adequate financing and resources is essential in relieving pressures of health service delivery.^1,30^

In addition, support from community leaders and social workers as well as community acceptance plays an important role in the success of the CHW programme. Evaluating the interaction of CHWs and the community from a cultural and gender perspective is therefore imperative; for example, male community members may resist interaction with female CHWs.^31^ Gender inequalities have also been shown to negatively impact HIV counselling and testing, HIV status disclosure and ART adherence, as well as the ability of female CHWs to work with male community members.^32^ In this regard, the introduction of male CHWs may be necessary to address these gender-related challenges and may result in improvement in health outcomes of male community members.^33^ However, the introduction of male CHWs may be thwarted by existing norms of hegemonic masculinity, where male CHWs assert dominance over their clients, limiting male community member’s ability to be open about being sick and vulnerable.^34^ This could also result in female community members feeling intimidated and withdrawn. When making these decisions to introduce male CHWs into the programme, it is imperative to adopt an approach that is conducive to developing supportive relationships between CHWs and community members that improve health-seeking behaviour.^34^

#### Challenging work environments and operational barriers

Minimal, inconsistent or irregular payment of stipends remains a critical challenge in maintaining CHWs interest and involvement in community-based programmes.^7,28^ Common reasons for CHWs resigning include inadequate compensation for their time, and family members being unsupportive of their workload in light of the minimal incentive received.^23,35^ Limited practical resources can also inhibit the effectiveness of CHW programmes. For example, a lack of comfortable footwear and umbrellas to protect CHWs from harsh weather elements may prohibit them from conducting household visits. The fact the CHWs have not been provided with official uniforms or name tags may also present a problem with regard to identification and authority of the CHWs in the community and health systems. Funding constraints also mean that CHWs often lack resources to travel to households, meetings and training sessions.^28^ Evaluations of CHW programmes show that CHWs become overwhelmed by their broad range of tasks, resulting in poor performance and work overload. Hermann et al.^6^ note that CHWs with a large number of tasks tend to select a few that they perceive as the most important or feasible to prioritise.^6^ The tasks they prioritise may or may not align with programmatic goals. Therefore, clearly defined management and reporting channels are essential to ensure that WBOTs are effectively used in the South African HIV programme. Moreover, the use of mobile technology should be considered to assist CHWs with their tasks and to improve communication. Studies show that the use of mobile technology by CHWs to collect field-based health data, receive reminders and alerts, facilitate health education and conduct person-to-person communication improved quality of care, service efficiency and programme monitoring.^36,37^

### Human immunodeficiency virus programme challenges

#### Lack of knowledge and skills

In South Africa, the NDoH developed a curriculum for CHWs, with the ultimate goal of national certification.^38^ CHWs are currently trained on HIV prevention and treatment, including provision of health education, screening community members for health problems and referrals to local facilities, included in the phase one training. They are therefore expected to have adequate knowledge to do their work. However, sustaining and updating knowledge through continual training is a challenge.^28^ Continuous education and support is therefore a requirement to sustain quality of the programme. Phase two training is currently underway, which will address knowledge gaps.^38^

#### Socio-cultural barriers

In a context where HIV is stigmatised, CHWs may be seen as a marker of HIV or AIDS as they are known to support individuals who are HIV-positive. This, in turn, can arouse a feeling of stigmatisation in community members which may result in individuals refusing to talk to CHWs, providing incorrect contact information, pretending to be someone else when visited by CHWs and asking to change the location of CHW visits. For example, there has been a report of CHWs specifically providing ART adherence support, trying to conceal their identities when visiting households by refraining from wearing uniforms given to them by their NGOs and pretending to be selling various commodities.^31^ Implementing community awareness and education campaigns aimed at reducing HIV-related stigma may therefore be an important factor facilitating the effectiveness of WBOTs. This may enable individuals to feel more comfortable to provide accurate medical information to their CHWs and details regarding treatment options can be shared with them by CHWs. This will also enable individuals to build relationships with external sources of support such as CHWs and PHC facilities in order to obtain healthcare advice and treatment.^31^ A formal WBOT policy is still in the process of being finalised. The policy may include ethical guidelines governing their roles, such as the requirement of registration with health bodies (currently not required), ethics training and the legal framework in which the CHWs operate.^38^ The policy may also stipulate the extent to which CHWs are qualified to provide health advice. This may improve CHWs credibility in the community.

## Perspective

There is great potential for CHWs to have a substantial impact on HIV programmes as evidenced above. We have presented several examples of successful CHW initiatives. CHW efforts can lead to improved health outcomes by improving community access to PHC in terms of health education, patient linkage to and retention in care. Expanding the CHW programme will further increase the potential to address large healthcare gaps. However, health system barriers such as human resource capacity, inconsistent remuneration, lack of nationally recognised training and a failure to mainstream WBOTs are important factors that limit the effectiveness of the WBOT programme. These need to be addressed for WBOTs to have maximum impact on the HIV and other health programmes.^23^ A particular point of concern is that CHWs may be overloaded with expectations and activities. In that regard, it is essential that the workforce size and quality of services provided must match the programme deliverables. Further investment in the WBOT programme would be essential to expand it; however, this is not easily achieved without a broad-scale documented impact. To our knowledge, there are no comprehensive data available on the impact and contribution of the WBOT structure on HIV programme outcomes in South Africa. Such impact would likely be highest in rural settings with high poverty and low education rates, but at the same time the operational barriers may be larger in those areas.^23^ Several initiatives are currently underway to document the impact that CHWs have on health programmes in general, and the HIV programme in particular, as well as to assess practical ways to improve their contribution. Community healthcare provides an important component of the South African healthcare system with high promise to support the usually overcrowded healthcare facilities, but strengthening of the structure is warranted to obtain maximum impact.

## Conclusion

There is evidence that WBOTs have a positive impact on healthcare. However, despite the strong potential, there are limited data on the impact of WBOTs on the South African HIV programme. Therefore, evaluations to determine operational barriers and opportunities for improvement are urgently warranted. This will provide much needed information to stakeholders regarding areas that need improvement and strengthening in order for WBOTs to become a sustainable structure in the South African healthcare system.
